# A Common Variant in the Telomerase RNA Component Is Associated with Short Telomere Length

**DOI:** 10.1371/journal.pone.0013048

**Published:** 2010-09-27

**Authors:** Omer T. Njajou, Elizabeth H. Blackburn, Ludmila Pawlikowska, Massimo Mangino, Coleen M. Damcott, Pui-Yan Kwok, Timothy D. Spector, Anne B. Newman, Tamara B. Harris, Steven R. Cummings, Richard M. Cawthon, Alan R. Shuldiner, Ana M. Valdes, Wen-Chi Hsueh

**Affiliations:** 1 Department of Medicine, University of California San Francisco, San Francisco, California, United States of America; 2 Department of Anesthesia and Perioperative Care, University of California San Francisco, San Francisco, California, United States of America; 3 Department of Biochemistry and Biophysics, University of California San Francisco, San Francisco, California, United States of America; 4 Cardiovascular Research Institute, University of California San Francisco, San Francisco, California, United States of America; 5 Twin Research and Genetic Epidemiology Unit, King's College London, St Thomas Hospital, London, United Kingdom; 6 Division of Endocrinology, Diabetes and Nutrition, Department of Medicine, University of Maryland, Baltimore, Maryland, United States of America; 7 Department of Epidemiology, University of Pittsburgh, Pittsburgh, Pennsylvania, United States of America; 8 Laboratory of Epidemiology, Demography, and Biometry, National Institute on Aging, Bethesda, Maryland, United States of America; 9 Research Institute, California Pacific Medical Center, San Francisco, California, United States of America; 10 Department of Human Genetics, University of Utah, Salt Lake City, Utah, United States of America; Ohio State University Medical Center, United States of America

## Abstract

**Background:**

Telomeres shorten as cells divide. This shortening is compensated by the enzyme telomerase. We evaluated the effect of common variants in the telomerase RNA component (*TERC*) gene on telomere length (TL) in the population-based Health Aging and Body Composition (Health ABC) Study and in two replication samples (the TwinsUK Study and the Amish Family Osteoporosis Study, AFOS).

**Methodology:**

Five variants were identified in the *TERC* region by sequence analysis and only one SNP was common (rs2293607, G/A). The frequency of the G allele was 0.26 and 0.07 in white and black, respectively. Testing for association between TL and rs2293607 was performed using linear regression models or variance component analysis conditioning on relatedness among subjects.

**Results:**

The adjusted mean TL was significantly shorter in 665 white carriers of the G allele compared to 887 non-carriers from the Health ABC Study (4.69±0.05 kbp vs. 4.86±0.04 kbp, measured by quantitative PCR, p = 0.005). This association was replicated in another white sample from the TwinsUK Study (6.90±0.03 kbp in 301 carriers compared to 7.06±0.03 kbp in 395 non-carriers, measured by Southern blots, p = 0.009). A similar pattern of association was observed in whites from the family-based AFOS and blacks from the Health ABC cohort, although not statistically significant, possibly due to the lower allele frequency in these populations. Combined analysis using 2,953 white subjects from 3 studies showed a significant association between TL and rs2293607 (β = −0.19±0.04 kbp, p = 0.001).

**Conclusion:**

Our study shows a significant association between a common variant in *TERC* and TL in humans, suggesting that *TERC* may play a role in telomere homeostasis.

## Introduction

Telomerase is a ribonucleoprotein polymerase that maintains telomere ends by addition of the telomere DNA repeat TTAGGG in humans. The core enzyme consists of a protein component with reverse transcriptase activity (*TERT*), and an RNA component (*TERC*) located on chromosome 3q26.2 that provides the template for the telomere repeat. Several lines of evidence in experimental and epidemiological research suggest that the human telomerase RNA component is involved in telomere homeostasis [1], [2], [3]. The wild-type telomerase RNA is assembled with small nucleolar RNAs (snoRNA), small Cajalbody RNA (scaRNA) proteins, the putative CAB box binding protein and telomerase reverse transcriptase to form a functional telomerase complex that will later be recruited to the chromosome ends and maintain telomere length [4].

*TERC* is a small gene, 451 basepairs (bp) long, that is highly expressed in the germline and in tumor cell lines, in which there is high telomerase activity, and at lower levels in tissues such as kidney, prostate, and liver in which there is little detectable telomerase activity. Mutations in *TERC* result in a reduction of telomerase activity leading to premature telomere shortening and have been linked to the autosomal dominant form of dyskeratosis congenita and aplastic anemia [5], [6]. Goldman et al. have observed that individuals haploinsufficient for *TERC* have very short telomeres [7]. They found that when *TERC* activity is limiting, this leads to the accelerated shortening of telomeres. The limited amount of active telomerase in individuals with *TERC* RNA haploinsufficiency may not be able to maintain the minimal critical telomere length in cells with already short telomeres. A recent study reported that a common haplotype in the other telomerase component, *TERT,* is associated with longer telomere length in centenarians [8].

Following previous studies of rare mutations in *TERC*, this study aimed at investigating whether common polymorphisms in or around the TERC gene region may play a role in the regulation of telomere length (TL) in normal populations.

## Materials and Methods

### Study populations

Three independent samples were used to investigate the association between common variants in or around *TERC* and TL. The first population is a biracial cohort (41% black and 59% white) from the Health, Aging, and Body Composition (Health ABC) Study. As we observed a significant association in the white samples only, we sought to replicate our initial findings in another outbred white population from the TwinsUK Study and a white founder population from the Amish Family Osteoporosis Study (AFOS).

### The Health ABC Study

The Health ABC Study population is a community-based cohort of 3,075 healthy, well functioning men and women aged 70 to 79 years. To be eligible for participation in the study, subjects had to report no difficulty in walking one-quarter mile (0.5 kilometers) or climbing 10 stairs without resting. Participants were identified from a random sample of white Medicare beneficiaries and all age-eligible black residents in designated zip code areas surrounding Pittsburgh and Memphis. Exclusion criteria included reported difficulty performing basic activities of daily living, obvious cognitive impairment, inability to communicate with the interviewer, intention of moving within three years, or participation in a trial involving a lifestyle intervention. A total of 2,620 subjects with TL measures and the *TERC* genotypic data available were included in this study. All participants gave written informed consent. The Committee on Human Research at both study sites approved the protocol and written consent for the study.

### The TwinsUK Study

The TwinsUK Study recruited white monozygotic (MZ) and dizygotic (DZ) twin pairs from the TwinsUK adult twin registry, a group designed to study the heritability and genetics of age-related diseases (www.twinsuk.ac.uk). These twins were recruited from the general population through national media campaigns in the UK and shown to be comparable to age-matched population singletons in terms of clinical phenotype and lifestyle characteristics. A subset of 696 female subjects with TL measures and the *TERC* genotypic data were included in this study. The study was approved by St. Thomas' Hospital Research Ethics Committee and all twins provided informed written consent that was approved by the Ethics Committee.

### The Amish Family Osteoporosis Study

A total of 954 Old Order Amish subjects for this study were recruited through the Amish Research Clinic in Strasburg, PA, as part of the Amish Family Osteoporosis Study (AFOS), whose aim was to identify genetic determinants of osteoporosis. The recruitment methods and study objectives and design have been described in details previously [9]. Briefly, individuals with low bone mineral density or history of fracture were recruited into the study as probands (n* = *57). Their spouses and all first-degree relatives aged 20 years and over were invited to participate in the study. Genealogical information was obtained from the Fisher Family History and the larger Anabaptist Genealogy Database version 3.0 [10], [11]. The protocol for the AFOS was approved by the Institutional Review Board of the University of Maryland, and verbal informed consent as well as permission to contact relatives was obtained from all participants. (Amish people do not use signature, so only verbal consent was given and this was approved by the review board).

### Measurement of leukocyte telomere length in the Health ABC Study and AFOS samples

The mean TL in leukocytes (peripheral blood mononucleocytes) was measured using a validated quantitative PCR (Q-PCR) method [12], [13] which measures the relative average TL in genomic DNA by determining the ratio of telomere repeat copy number to single copy gene copy number (T/S ratio) in experimental samples relative to a reference sample. The T signal for an experimental DNA sample is the number of nanograms of the reference DNA that matches the experimental sample for copy number of the telomere repeats. The S signal is the number of nanograms of the reference DNA that matches the experimental sample for copy number of the single copy gene. Experimental samples with T/S ratio >1.0 have longer average telomere lengths than the reference DNA. Experimental samples with T/S ratio <1.0 have shorter average telomere lengths than the reference DNA. The reference DNA is a pooled sample of DNAs from several normal Utah whites, aged 65 years or older. All samples were measured in triplicate and the mean value was used. The coefficient of variation (CV) for this assay was 4%. Results obtained using this method correlate very well with those obtained with the traditional terminal restriction fragment length (TRFL) by Southern blot technique [12]. In comparison with the TRFL method, the Q-PCR method is simple, fast, and less expensive, and requires a significantly lower amount of DNA. To convert the T/S value to basepairs, the T/S value was multiplied by a conversion factor of 4,270 bp, which was the TL for the reference DNA. To obtain the TL for the reference DNA, we used the T/S ratios of 64 DNA samples with known mean TRF lengths. The slope of the linear regression line through a plot of T/S ratio (the X axis) vs. mean TRF length (the Y axis) is the number of basepairs of telomeric DNA corresponding to a single T/S unit. Since the reference DNA has a T/S of 1.0, by definition, this slope is also the average TL of the reference DNA sample, 4,270 bp in our case. Among the 3,075 participants at baseline, 2,880 subjects had DNA available and TL was successfully measured in 2,721 individuals.

### Measurement of leukocyte telomere length in the TwinsUK Study samples

The mean leukocyte TL was assessed using the TRFL method, which was measured using the Southern blot method in duplicates. Briefly, each sample was digested using restriction enzymes *Hinf*I (10 U) and *Rsa*I (10 U) (Roche, Indianapolis, Indiana, USA) and resolved on 0.5% agarose gels. DNA was then depurinated and denatured. Hybridization with digoxenin 3′-end-labeled telomeric probes was conducted overnight and probes detected using a digoxenin luminescent detection procedure (Roche). The autoradiographs were scanned and the TRFL signal was digitized at molecular weight 1–20 kb. Conversion of the optical density versus DNA migration distance to optical density adjusted for background/molecular weight yielded a histogram from which the mean TRFL was calculated. The CV of the TRFL assay in this study was 1.4%.

The laboratories conducting the telomere length measurements were blinded to all characteristics of the leukocyte donors, who were identifiable only by coded ID numbers.

### Discovery and genotyping of the *TERC* variants

Variants in *TERC* covering the entire gene of 451 bp plus 385 bp upstream and 265 bp downstream of the coding region were identified by conventional Sanger sequencing in pools of PCR products (8 pools of white and 8 pools of black, with 5 individuals per pool). Because no protein is encoded by *TERC*, the "coding" region refers to the sequence encoding the mature RNA transcript. Sequencing was performed using the forward primer and standard procedures on the ABI 3700 capillary sequencer. Sequencing traces and subsequent genotyping were scored by 2 investigators blinded to phenotype (telomere measurement status). Five SNPs were identified by sequence analysis and the allele frequencies were estimated from the trace heights in pools (see Table 1). The SNPs at base # 170,965,315 and 170,965,029 have been previously reported and correspond to base # 228 and 514 in those reports [14], [15]. The other three SNPs were newly discovered. We genotyped these five SNPs in the full Health ABC cohort by sequencing or by Illumina Golden Gate and subsequently we genotyped the only common SNP in the TwinsUK and the AFOS samples by the Taqman method.

**Table 1:** Sequence variants identified in the *TERC* gene.

Base #	Location	Base pair change	Minor allele frequency in white	Minor allele frequency in black	Sequence location
170,965,788	5′ upstream	G/A	0.01	0.01	tagaaaaaag[g/a]ccctctgata
170,965,485	RNA coding	G/A	0.04	0.10	aaccctaact[g/a]agaagggcgt
170,965,315	RNA coding	G/A	0	0.03	cgggtcgcct[g/a]cccagccccc
170,965,029 (rs2293607)	3′ flank	A/G	0.30	0.05	ctcgccggca[a/g]tgggggcttg
170,964,909	3′ flank	G/A	0.01	0	ggggttgcct[g/a]gagccgttcc

### Statistical analyses

The associations between leukocyte TL and common *TERC* SNPs were modeled using linear regression models. Age, sex, race, and the recruitment site were added to this model to control for potential confounding. To account for the relatedness of twins in the TwinsUK cohort, we used mixed linear models where the random effect was the family of origin. Analyses for the Health ABC and UK cohort data were carried out using the software SPSS 14 for Windows (Chicago, IL) and analyses for the AFOS cohort were conditioning on their relatedness and carried out using the program SOLAR [16]. Since significant associations were observed only in white, we also conducted combined analysis by pooling data from all 3 white populations. The combined analysis was carried out in SOLAR so that the relatedness among subjects (in the TwinsUK and AFOS cohorts) can be taken into account. In addition to covariates used in population-specific analysis, the type of TL assay was also included as a covariate. A p value <0.05 was considered statistically significant.

## Results

Of the five variants identified by sequence analysis of the *TERC* gene region, only one SNP (i.e. rs2293607, G/A) had a minor allele frequency (MAF) >5% (see Table 1). The frequency of the G allele was 0.12–0.26 in 3 white populations, and was 0.074 in blacks (see Table 2). A total of 2,620 individuals (1,542 white and 1,078 black) from the Health ABC cohort, 696 female Caucasian participants of the TwinsUK cohort and 745 Caucasian subjects from the AFOS cohort were included in our analysis. Table 2 shows the baseline characteristics of the 3 study populations. Participants in the Health ABC Study were older (mean age  = 73.7±2.9 years) compared to those in the TwinsUK (mean age  = 48.6±13.4) and the AFOS (mean age  = 50.4±16.5) populations. However, the age range was much narrower in the Health ABC cohort compared to the other two cohorts comprised of younger participants. Nearly half of the Health ABC cohort and 62% of AFOS participants were female, while all participants of the TwinsUK cohort were female.

**Table 2:** Characteristics of study participants from three cohorts.

Traits (Mean ± SD or number, %)	Health ABC (White)	Health ABC (Black)	TwinsUK (White)	AFOS (White)
N (% female)	1,542 (48%)	1,078 (58%)	696 (100%)	745 (62%)
Age (years)	73.8±2.9	73.5±2.9	48.6±13.4	50.4±16.5
Range (years)	69–80	68–80	18–79	20–92
Telomere length (kbp)	4.8±1.2	4.9±1.2	7.0±0.7*	6.1±1.7
Range (kbp)	1.7–8.5	1.7–8.8	5.2–9.1	2.6–12.5
Frequency of the G allele of rs2293607	24.2%	7.4%	26.3%	11.5%
rs2293607 genotypes				
AA	887 (57.5%)	930 (86.3%)	395 (56.8%)	581 (78.0%)
AG	563 (36.5%)	136 (12.6%)	236 (33.9%)	157 (21.1%)
GG	92 (6.0%)	12 (1.1%)	65 (9.3%)	7 (0.9%)

*Including the subtelomeric segment.

Overall, the mean TL (kbp) measured by Q-PCR was longer among Amish subjects (6.1±1.7 kbp) compared to that of the Health ABC participants (4.9±1.2 kbp), but in the same age group (70–79 years), the mean TL in 2 populations were similar. The mean TL in the TwinsUK cohort measured by Southern blot appeared to be longer (7.0±0.7 kbp). The frequency of the G allele of rs2293607 was ∼25% in 2 outbred white populations while it was much rarer in the white founder population (the Amish, 11.5%) and black samples (7.4%). Table 3 shows the mean TL by rs2293607 genotypes. There was a significant difference in the mean TL by genotypes (2-df test) in the Health ABC white (p = 0.02) and the TwinsUK cohort (p = 0.05). A dose-response of TL with respect to the number of G alleles was observed in the Health ABC whites and the AFOS. Modeling of the association between TL and rs2293607 genotypes suggested that a dominant mode of inheritance for the minor G allele was the best fit to the data. As shown in Table 3, among the Health ABC Study white participants, the adjusted mean TL was significantly shorter in carriers of the G allele (4.69±0.05 kbp vs. 4.86±0.04 kbp in non-carriers, p<0.005). No significant difference was observed in blacks (4.85±0.10 kbp in carriers of the G allele vs. 4.88±0.04 kbp in non-carriers, p = 0.8). We successfully replicated the association in whites from the TwinsUK Study. The adjusted mean TL was significantly (p<0.009) shorter in carriers of the G allele compared to non-carriers. In the AFOS, carriers of the G allele appeared to have shorter TL on average compared to non-carriers, but the difference was not statistically significant.

**Table 3:** Association between telomere length and rs2293607.

Ethnicity					Caucasian						Black	
Study		Health ABC			AFOS			TwinsUK			Health ABC	
		N (%)	TL ± SE		N (%)	TL ± SE		N (%)	TL ± SE		N (%)	TL ± SE
**Means by genotype**												
	AA	887 (57.5)	4.86±0.04		581 (78.0)	6.95±0.15		395 (56.8)	7.07±0.03		930 (86.3)	4.88±0.04
	GA	563 (36.5)	4.69±0.05		157 (21.1)	6.69±0.25		236 (33.9)	6.90±0.04		136 (12.6)	4.88±0.12
	GG	92 (6.0)	4.65±0.10		7 (0.9)	6.25±0.11		65 (9.3)	7.06±0.09		12 (1.1)	4.53±0.34
P value			0.02			NS			0.05			NS
	AA	887 (57.5)	4.86±0.04		581 (78.0)	6.95±0.15		395 (56.8)	7.07±0.03		930 (86.3)	4.88±0.04
	GG + GA	655 (42.5)	4.69±0.05		164 (22.0)	6.67±0.17		301 (43.2)	6.93±0.03		148 (13.7)	4.85±0.10
P value>*			0.005			NS			0.009			NS
**Association** *												
β± SE			−0.17±0.05			−0.21±0.26			−0.11±0.05			−0.09±0.13
P value			0.005			NS			0.009			NS

*Based on a dominant genetic model for the G allele, adjusted for age, sex, relatedness, telomere length assay batch.

As the effect of rs2293607 appeared similar among 3 white study populations, we pooled data from all these populations (n = 2,983) and found that the mean TL for carriers of the minor G allele was 0.19 kbp shorter than non-carriers (β = −0.19±0.04 kbp, p<0.001).

## Discussion

We observed that the G allele of the SNP rs2293607 near *TERC* (63 bps upstream from the start site) was significantly associated with shorter telomere length in a US white population and replicated the findings in another younger white population in the UK. On the other hand, we did not observe such an association in whites from the family-based AFOS or in blacks from the Health ABC cohort. The G allele was rarer in the AFOS sample (frequency  = 11.5%) and in the blacks (7.4%) compared to whites from Health ABC Study (24.2%) or the TwinsUK Study (26.3%). Thus, the non-significant associations in these two populations are likely due to the lack of sufficient statistical power (we had only 55% power) to detect the same effect size observed in the Health ABC cohort.

A recently published genome-wide association study of telomere length by Codd et al. reported their top signal to be rs12696304, 1.5 kb downstream of *TERC* [17]. This SNP resides in the same LD block of 33 kb and is 1,066 bps away (NCBI build 36.3) from our reported SNP rs2293607. We looked into this region more closely for all typed SNPs in the 33 kb block, including rs12696304. All of these SNPs were significantly associated with telomere length, but rs2293607 still gave the strongest signal and is in high LD (r^2^ = 0.90) with rs12696304. The SNP rs2293607 is not typed by the Hapmap and therefore not included in any high-throughput genotyping platform. As this SNP is closer to the mature RNA transcript of TERC, it might be a better target for further functional studies. Both this report and the report by Codd et al. detected the RNA template of telomerase as major determinant for human telomere length variation. It is interesting that the yeast TERC ortholog, TLC1, is a major telomere length QTL in the natural population [18]. It does appear that this gene is prone to be a determinant for telomere length variation. This could be due to its dynamic structure or by differences in gene expression. Indeed, a number of telomerase RNA template molecules appear to be a limiting factor of telomere elongation in budding yeast. Similarly, there is a pronounced TERC haploinsufficiency effect in human telomeres and this agrees with our findings for the rs2293607 SNP.

One possible issue of our replication sample is that the TwinsUK study subjects consisted only of females. However, in the Health ABC cohort the reported association did not differ by sex. Another issue has to do with different methods for measuring TL. TL was measured by the Q-PCR method in the Health ABC and Amish cohorts while it was measured by the TRFL method in the TwinsUK Study. The TRFL method tends to make the TL about 2.5 kb longer as it includes the subtelomeric region in its measurement. Nevertheless, the strength of the association and the effect size estimates appeared comparable based on either method.

There have been debates regarding TL measurements as to whether different methods for TL measurement could affect the informativeness of TL measures for genetic and epidemiological studies involving TL [19], [20], [21]. Therefore, it is reassuring that we were able to replicate our observed association in the Health ABC Study (TL measured by the Q-PCR method) and in the TwinsUK Study (TL measured by the TRFL method). In addition, in the combined analysis adjusted for the method of TL measurement, the observed association remained significant and in the same direction.

Some environmental factors like smoking are found to affect TL [22]. In our study, TL was not associated with smoking and adjusting for smoking did not alter our observations. We however adjusted our analysis for the TL assay's plate effects which has been associated to TL measurements in several laboratories.

The TERC and TERT genes encode the core essential elements of the telomerase complex that replicates telomeres and maintains their length. Mutations in *TERC* have been previously linked to telomere shortening in mouse models [23]. Studies in human have also shown that rare mutations in *TERC* are associated with telomere shortening in individuals with dyskeratosis congenita [4], [7], [24]. Our study extends these observations to populations free of congenital or other severe diseases. The biological relevance of our observations is further supported by a recent report that variants in another telomerase RNA component, *TERT*, are also associated with TL in humans. Both *TERT* and *TERC* are essential for telomerase function. The *TERC* region does not appear to be conserved in evolution. There is an open reading frame (ORF) antisense to *TERC*. However, the likelihood that there is a protein encoded on either strand of the TERC locus is extremely remote, as indicated by codon usage/substitution patterns in the ORFs, the lack of peptides in mass spectrometry databases that map to these ORFS, and the lack of homology of any of the ORFS to any known protein sequence. There is not yet functional information on rs2293607 and it is beyond the scope of our current investigation. One possible functional study is to take advantage of the induced pluripotent stem (iPS) cells technique since *TERC* upregulation is a feature of the pluripotent state and several telomerase components are targeted by pluripotency-associated transcription factors [25]. Conventionally, genes that do not encode proteins are thought to be of little relevance with clinically measurable phenotypes. However, a growing number of human studies of RNA genes such as *TERC* [17] and microRNA genes [26] suggest that non-coding regions such as these RNA genes may harbor genetic variants of clinical relevance.

Taken together, our findings suggest that common variants in the TERC gene in normal healthy populations may be involved in telomere homeostasis. Our observations further re-enforce the idea that variants nearby genes encoding functional RNAs may potentially be biologically important.
